# Prognostic value of localization of epidermal growth factor receptor in lung adenocarcinoma

**DOI:** 10.1186/s12929-018-0451-3

**Published:** 2018-06-28

**Authors:** Jinn-Li Wang, Chia-Lang Fang, Yu-Tien Tzeng, Han-Lin Hsu, Sey-En Lin, Ming-Chih Yu, Kuan-Jen Bai, Liang-Shun Wang, Hsingjin Eugene Liu

**Affiliations:** 1 Division of Hematology Oncology, Department of Pediatrics, Wan Fang Hospital, Taipei Medical University, No.111, Sec. 3, Xinglong Rd, Wenshan Dist, 11696 Taipei, Taiwan; 2 Department of Pediatrics, School of Medicine, College of Medicine, Taipei Medical University, 250 Wuxing St. Taipei, 11031 Taipei, Taiwan; 3 Graduate Institute of Clinical Medicine, Collage of Medicine, Taipei Medical University, 250 Wuxing St. Taipei, 11031 Taipei, Taiwan; 4 Department of Pathology, Wan Fang Hospital, Taipei Medical University, No.111, Sec. 3, Xinglong Rd, Wenshan Dist, 11696 Taipei, Taiwan; 5 Department of Pathology, School of Medicine, College of Medicine, Taipei Medical University, 250 Wuxing St. Taipei, 11031 Taipei, Taiwan; 6 Division of Pulmonary Medicine, Department of Internal Medicine, Wan Fang Hospital, Taipei Medical University, No.111, Sec. 3, Xinglong Rd, Wenshan Dist, 11696 Taipei, Taiwan; 7 Graduate Institute of Medical Sciences, College of Medicine, Taipei Medical University, 250 Wuxing St. Taipei, 11031 Taipei, Taiwan; 8 Division of Thoracic Surgery, Department of Surgery, Shuang Ho Hospital, Taipei Medical University, No.291, Zhongzheng Rd., Zhonghe District, New Taipei City, 23561 Taiwan; 9 Division of Hematology Oncology, Department of Internal Medicine, Wan Fang Hospital, Taipei Medical University, No.111, Sec. 3, Xinglong Rd, Wenshan Dist, 11696 Taipei, Taiwan

**Keywords:** Epidermal growth factor receptor, Lung adenocarcinoma, Survival, Immunohistochemistry, Localization

## Abstract

**Background:**

The nuclear translocation of epidermal growth factor receptor (EGFR) has been considered to play a role in carcinogenesis. However, the relevance of differentially located EGFR proteins in lung cancer remains unclear.

**Methods:**

We examined 161 patients with primary lung adenocarcinoma to detect EGFR expression in lung cancer cells using immunohistochemistry and determined the correlations of EGFR expression with clinical characteristics, EGFR mutations, and survival time. Moreover, we graded complete membranous staining with strong intensity as high membranous EGFR (**m**EGFR) expression, and nuclear EGFR staining with strong intensity as high nuclear (**n**EGFR) expression.

**Results:**

The prevalence of high **m**EGFR and **n**EGFR expression in lung adenocarcinoma was 42.86 and 39.13%, respectively. After multivariate analyses, high **m**EGFR expression was associated with a significantly reduced mortality risk in older patients, those with a history of smoking, and those without brain metastasis (hazard ratio[95% confidential interval], HR[95% CI] = 0.55[0.32~ 0.92]; 0.51[0.26~ 0.98] and 0.56[0.33~ 0.94], in overall survival, respectively). An association between high **n**EGFR expression and early recurrence was observed in patients with metastasis (HR[95% CI] =1.68[1.05~ 2.68], in progression-free survival). Notably, patients with low **m**EGFR and low **n**EGFR expression had the lowest survival rate in cases without brain metastasis (*p* = 0.018) and with a history of smoking (*p* = 0.062) and total EGFR (any high **m**EGFR or **n**EGFR) expression indicated a more favorable response to platinum-based chemotherapy regardless of EGFR mutations (HR[95% CI] =0.33[0.12–0.92]; adjusted HR[95% CI] = 0.36[0.13~ 1.02] with the use of tyrosine kinase inhibitor).

**Conclusions:**

EGFR proteins at different cellular locations in lung adenocarcinoma might influence the biology of cancer cells and are an independent indicator of more favorable prognosis and treatment response.

**Electronic supplementary material:**

The online version of this article (10.1186/s12929-018-0451-3) contains supplementary material, which is available to authorized users.

## Background

Lung cancer is the leading cause of deaths worldwide, including in Taiwan. The 5-year overall survival (OS) rate of advanced-stage lung cancer is less than 20% [1]. Tyrosine kinase inhibitors (TKIs, erlotinib or gefitinib) have been prescribed in lung adenocarcinoma patients who have epidermal growth factor receptor (EGFR) mutations and have markedly improved the survival outcome, but patients still eventually develop TKI resistance [2, 3]. Thus, a more comprehensive understanding of lung carcinogenesis is necessary to develop more effective therapies.

Overexpression of EGFR is implicated in the pathogenesis of many human malignancies, including lung cancer [4]. EGFR overexpression has been reported to be strongly associated with cancer progression and to predict shorter survival in surgically resected non-small cell lung cancer (NSCLC) [5, 6]. Nevertheless, high EGFR expression may predict the response to gefitinib in lung adenocarcinoma with a high survival and provide survival benefits when gefitinib is used in combination with cetuximab in advanced NSCLC with wildtype EGFR status [7–9]. Therefore, a more detailed understanding of EGFR biology in lung carcinomas is required.

The localization of nuclear EGFR, which has been detected in various cancers in the last decade, functions as a transcription factor for cell proliferation, angiogenesis and resistance to standard therapy [10]. Nuclear EGFR expression has been reported to be related to disease progression and poor survival time in breast, ovary and oropharynx cancers as well as in early stage NSCLC [11–14]. However, few studies investigate the relationship of between EGFR proteins and EGFR mutations [15, 16].

Since the clinical correlation of differentially located EGFR proteins in lung cancer has not been completely evaluated; therefore, we intended to investigate the relevance of differentially located EGFR expression in lung adenocarcinoma. This study retrospectively graded differentially located EGFR expressions in 161 lung adenocarcinoma specimens by using immunohistochemistry (IHC) and determined the association with demographic characteristics, stages, EGFR mutation status, and survival time.

## Methods

### Patients and tumor specimens

Formalin-fixed paraffin-embedded tumors from 161 patients with lung adenocarcinoma diagnosed based on 2015 WHO classifications [17], who had undergone computed tomography-guided needle biopsies or wedge resections were collected from Taipei Medical University-Wan Fang Hospital between 2008 and 2014. The clinical parameters and follow-up data were obtained by reviewing medical records. The patients were followed until December 2016; the median follow-up period was 13.1 months (0.07–132.03). All survivors were followed for at least 12 months. This study was approved by the Joint Institutional Review Board of Taipei Medical University.

### Immunohistochemistry

Four-micrometer sections of paraffin-embedded blocks were deparaffinized in xylene substitute, rehydrated with alcohol, and subjected to antigen retrieval. To detect EGFR proteins in the different cellular compartments of cancer cells, we used two types of primary EGFR antibodies to recognize EGFR proteins by the specific terminus. The mouse anti-EGFr antibody (clone 31G7, Invitrogen, Breda, the Netherlands) is raised against the NH2 terminus and recognizes membranous EGFR (**m**EGFR) and cytoplasmic EGFR [18]; the NCL-EGFR-384 antibody (clone EGFR.25, Novocastra, Newcastle, upon Tyne, UK) is raised against the COOH terminus and recognizes both **m**EGFR and nuclear EGFR (**n**EGFR) [11, 19]. Appropriate antigen retrieval protocols were used according to the specific primary antibodies. For **m**EGFR protein detection, we used the enzyme digestion method with pepsin (Digest-All™ 1, Thermo Fisher Scientific) for 10 min at 37 °C and for **n**EGFR protein detection, we used the double-antigen retrieval method, heat-induced epitope retrieval (boiling citrate buffer for 5 min) plus enzyme digestion with 0.025% trypsin **(**T4799, Sigma Aldrich) for 8 min at room temperature (RT). Slides were incubated at RT with the mouse anti-EGFr antibody overnight and at 4 °C with the NCL-EGFR-384 antibody overnight. The labeled streptavidin biotin method with horse radish peroxidase was used to achieve signal amplifications. Immunoreactions were visualized using 3.3′-diamino-benzidine-tetrahydrochloryte, then counterstained with hematoxylin.

### IHC interpretation

**Fig. 1:**
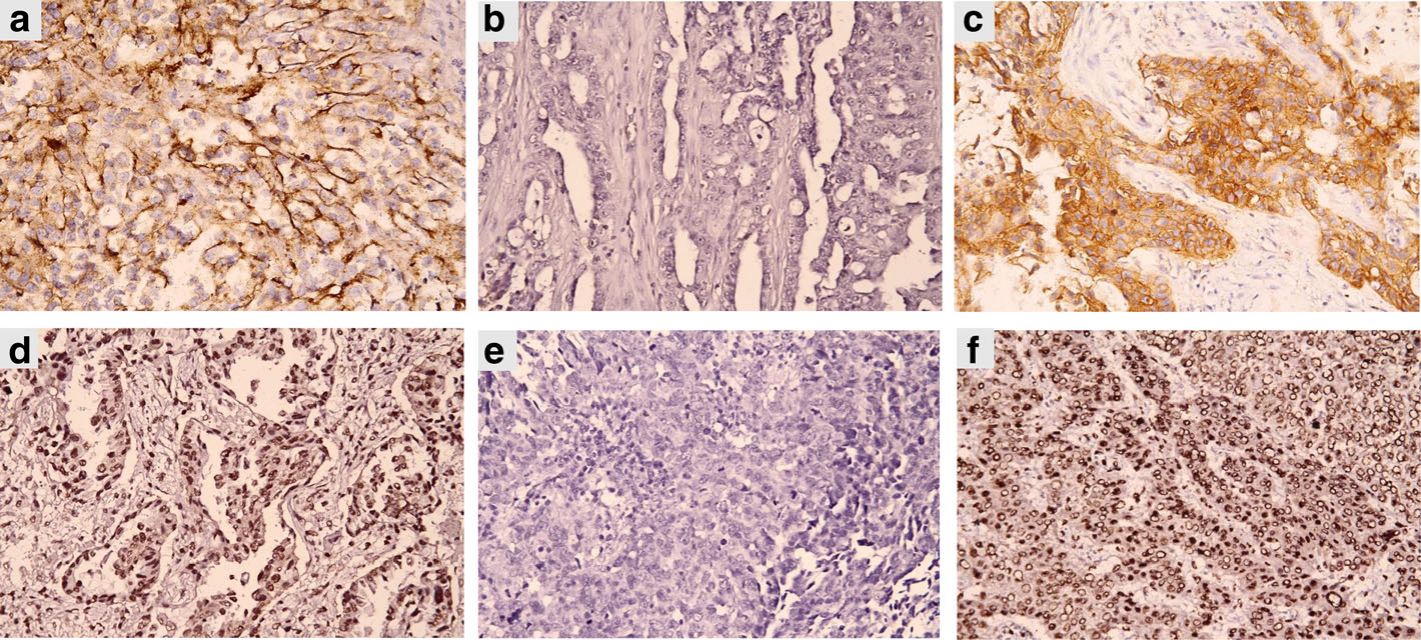
Immunostaining for **m**EGFR and **n**EGFR proteins in lung adenocarcinoma tissues. **a** high **m**EGFR expression; **b** low **m**EGFR expression; **c** positive **m**EGFR control (lung squamous cell carcinoma); **d** high **n**EGFR expression; **e** low **n**EGFR expression; **f** positive **n**EGFR control (hepatocellular carcinoma). Original magnification × 400

**m**EGFR and **n**EGFR expression was interpreted as high and low expression from IHC images. We graded complete membranous staining with strong intensity in more than 10% of cancer cells as high **m**EGFR expression and others (incomplete membranous, weak or less than 10% of cancer cells staining) as low **m**EGFR expression. Nuclear staining with strong intensity in more than 10% of cancer cells was graded as high **n**EGFR expression and others were as low **n**EGFR expression. Lung squamous cell carcinoma tissue, known to have **m**EGFR overexpression, served as the positive control for **m**EGFR staining and hepatocellular carcinoma tissue served as the positive control for **n**EGFR staining. The negative controls of **m**EGFR and **n**EGFR comprised slides with the mouse IGG1 isotype antibody. The immunostain grading was done by two designated pathologists (C-L. F. and S-E. L.) with total agreement blindly. Figure 1 shows representative images for **m**EGFR and **n**EGFR expression.

### Statistical analyses

The Pearson chi-squared test was performed to analyze the associations between differentially located EGFR proteins and clinical characteristics. The Kaplan-Meier estimate by using the log-rank test was employed to evaluate the survival distributions for differentially located EGFR proteins. OS was defined as the time between the date of diagnosis and that of death from any causes or the date of censorship (date of final follow-up). Progression-free survival (PFS) was defined as the time between the date of treatment initiation to that of tumor progression, death from any causes, or censorship. To evaluate the mortality risk, the hazard ratio (HR) and corresponding confidence interval (CI) were estimated using Cox proportional hazards models to identify potential prognostic factors. All statistical analyses were conducted using SAS version 9.4 and as *p* < 0.05 was considered statistically significant.

## Results

### Distribution of differentially located EGFR proteins in cancer tissues

Among the 161 lung adenocarcinoma specimens, high **m**EGFR and **n**EGFR expression was observed in 69 (42.86%) and 63 (39.13%) specimens, respectively. The distribution of differentially located EGFR proteins in cancer tissues had shown 36 (22.36%) with high **m**EGFR and **n**EGFR staining, 33 (20.50%) with high **m**EGFR and low **n**EGFR staining, 27 (16.77%) with low **m**EGFR and high **n**EGFR staining, and 65 (40.37%) with low **m**EGFR and **n**EGFR staining. **m**EGFR expression significantly correlated with **n**EGFR expression (*p* = 0.0033, Additional file 1: Table S1). In addition, we found that morphologic characteristics were different in cancer tissues with high **m**EGFR expression, high **n**EGFR expression and EGFR mutations. The case numbers in certain subtypes of pathology were too small to draw definite conclusion. The detailed data was shown in Additional file 1: Table S2.

### Clinical significance of different localizations of EGFR protein

A significantly high number of patients with low nodal stage had high **m**EGFR expression (odds ratio[OR, 95% CI] = 2.01[1.06–3.81], *p* = 0.031; adjusted OR[95% CI] = 3.92[1.25~ 12.27], *p* = 0.019, respectively, Table 1). Compared with those with low **m**EGFR expression, high **m**EGFR expression was significantly associated with low recurrence risk in patients without brain metastasis (in univariate analyses, HR[95% CI] = 0.63[0.40–0.99], *p* = 0.045; in multivariate analyses, HR[95% CI] = 0.56 [0.34~ 0.91], *p* = 0.018 in PFS) and was significantly associated with reduced mortality risk in older patients (> 70 years), those with a history of smoking, and those without brain metastasis (in univariate analyses, HR[95% CI] = 0.59[0.36–0.97], 0.54[0.30–0.99] and 0.56[0.34–0.91] in OS, respectively; in multivariate analyses, 0.55[0.32~ 0.92], 0.51[0.26~ 0.98] and 0.56[0.33~ 0.94] in OS, respectively). High **n**EGFR expression was significantly associated with recurrence risk (in univariate analyses, HR[95% CI] = 1.58[1.01–2.45] in PFS and in multivariate analyses, HR[95% CI] = 1.68[1.05~ 2.68] in PFS, respectively), but did not affect mortality risk (*p* = 0.677). Notably, patients with a history of smoking who had high **n**EGFR expression had a significantly lower mortality risk than those who had low **n**EGFR expression (in univariate analyses, HR[95% CI] = 0.55[0.30–0.99] in OS and in multivariate analyses, HR[95% CI] = 0.49[0.25~ 0.97] in OS, respectively). All data are presented in Tables 2 and 3. However, EGFR expressions (**m**EGFR and **n**EGFR) were not affected by EGFR mutations (*p* = 0.205 and *p* = 0.734, Table 1).

**Table 1:** Characteristics of high membranous and nuclear EGFR expression in lung adenocarcinoma

Characteristics	No	High membranous EGFR (*N* = 69)	OR (95% CI)	*P* value	High nuclear EGFR (*N* = 63)	OR (95% CI)	*P* value
Age (median = 71 yr)				0.423			0.966
Younger	77	30 (39.0)^a^	1		30 (38.9)	1	
Older > 70 yr	84	39 (46.4)	1.36 (0.73~ 2.54)		33 (39.3)	1.01 (0.54~ 1.91)	
Gender				0.436			0.692
Female	90	41 (45.6)	1		34 (37.8)	1	
Male	71	28 (39.5)	0.78 (0.41~ 1.46)		29 (40.8)	1.14 (0.60~ 1.46)	
Smoking				0.776			0.919
Never	103	58 (56.3)	1		40 (38.8)	1	
Current or past	58	34 (58.6)	0.91 (0.47~ 1.75)		23 (39.7)	1.04 (0.54~ 1.20)	
Tumor stage (2 missing)				0.584			0.455
T1/T2	80	33 (41,3)	1		34 (42.5)	1	
T3/T4	79	36 (45.6)	1.19 (0.64~ 2.23)		29 (36.7)	0.78 (0.42~ 1.48)	
Nodal stage (1 missing)				**0.031**			0.466
L0/L1	68	36 (52.9)	2.01 (1.06~ 3.81)^b^		29 (42.6)	1.27 (0.67~ 2.41)	
L2/L3	92	33 (35.9)	1		34 (36.9)	1	
Metastasis				0.090			0.118
without	67	34 (50.7)	1		31 (46.3)	1	
with	94	35 (37.2)	0.58 (0.30~ 1.09)		32 (34.0)	0.60 (0.32~ 1.14)	
TNM stage				**0.045**			0.247
Localized (stage I/II)	43	24 (55.8)	2.05 (1.01~ 4.16)^c^		20 (46.5)	1.52 (0.75~ 3.08)	
Distant (stage III/IV)	118	45 (38.1)	1		43 (36.4)	1	
Brain metastasis				0.515			0.890
without	114	47 (41.2)	1		45 (39.5)	1	
with	47	22 (46.8)	1.25 (0.63~ 2.49)		18 (38.3)	0.95 (0.47~ 1.91)	
EGFR mutations^d^				0.205			0.634
Wildtype	74	28 (37.8)	1		27 (36.5)	1	
Mutations^e^	77	37 (48.0)	1.52 (0.79~ 2.91)		31 (40.3)	1.17 (0.61~ 2.26)	

Chi-squared test

^a^Data are presented as n (%) of row

^b^Adjusted OR (95% CI) = 3.92 (1.25~ 12.27), *p* = 0.019

^c^Adjusted OR (95% CI) = 1.37 (0.32~ 5.98) *p* = 0.674

^d^10 missing

^e^Including 3 in exon 18, 37 in exon 19, 3 in exon 20, 33 in exon 21and 1 in exon 19/20

Note: Boldfaces as statistical significance

**Table 2:** Univariate analyses for progression-free and overall survival in lung adenocarcinoma

Variable		High membranous EGFR				High nuclear EGFR			
Parameter	No	PFS		OS		PFS		OS	
		^a^HR (95%CI)	*p*	^a^HR (95%CI)	*p*	^b^HR (95%CI)	*p*	^b^HR (95%CI)	*p*
All patients	161	0.79 (0.56~ 1.13)	0.198	0.71 (0.49~ 1.03)	0.067	1.03 (0.73~ 1.46)	0.865	0.81 (0.56~ 1.17)	0.251
Older age (> 70 yr)	84	0.64 (0.39~ 1.04)	0.068	0.59 (0.36~ 0.97)	**0.038**	0.81 (0.50~ 1.31)	0.394	0.69 (0.40~ 1.10)	0.101
Smokers	58	0.62 (0.35~ 1.09)	0.092	0.54 (0.30~ 0.99)	**0.046**	0.61 (0.34~ 1.10)	0.102	0.55 (0.30~ 0.99)	**0.043**
Distant (metastasis)	94	1.01 (0.66~ 1.56)	0.954	0.83 (0.54~ 1.29)	0.403	1.58 (1.01~ 2.45)	**0.043**	1.10 (0.70~ 1.73)	0.677
No brain metastasis	114	0.63 (0.40~ 0.99)	**0.045**	0.56 (0.34~ 0.91)	**0.019**	1.01 (0.66~ 1.56)	0.953	0.78 (0.49~ 1.25)	0.297
Wildtype EGFR	74	0.60 (0.35~ 1.03)	0.059	0.64 (0.37~ 1.09)	0.094	0.84 (0.50~ 1.41)	0.506	0.74 (0.43~ 1.26)	0.265

Cox proportional hazards model

^a^Low membranous EGFR expression as a reference

^b^Low nuclear EGFR expression as a reference

Note: Boldfaces as statistical significance

**Table 3:** Multivariate analyses for progression-free and overall survival in lung adenocarcinoma

Variable		High membranous EGFR				High nuclear EGFR			
Parameter	No	PFS		OS		PFS		OS	
		^a^HR (95%CI)	*p*	^a^HR (95%CI)	*p*	^b^HR (95%CI)	*p*	^b^HR (95%CI)	*p*
All patients	161	0.72 (0.50~ 1.04)	0.078	0.70 (0.47~ 1.03)	0.071	0.89 (0.61~ 1.29)	0.529	0.72 (0.49~ 1.07)	0.103
Older age (> 70 yr)	84	0.60 (0.36~ 0.99)	0.046	0.55 (0.32~ 0.92)	**0.023**	0.77 (0.47~ 1.27)	0.310	0.62 (0.37~ 1.05)	0.077
Smokers	58	0.63 (0.34~ 1.16)	0.135	0.51 (0.26~ 0.98)	**0.045**	0.64 (0.34~ 1.21)	0.166	0.49 (0.25~ 0.97)	**0.041**
Distant (metastasis)	94	1.20 (0.76~ 1.88)	0.434	1.00 (0.63~ 1.61)	0.985	1.68 (1.05~ 2.68)	**0.030**	1.27 (0.77~ 2.08)	0.350
No brain metastasis	114	0.56 (0.34~ 0.91)	**0.018**	0.56 (0.33~ 0.94)	**0.028**	0.86 (0.54~ 1.36)	0.521	0.72 (0.44~ 1.18)	0.186
Wildtype EGFR	74	0.49 (0.28~ 0.85)	**0.012**	0.51 (0.29~ 0.90)	**0.019**	0.73 (0.43~ 1.24)	0.240	0.62 (0.36~ 1.08)	0.092

Cox proportional hazards model after adjustment of other variables except distant

^a^Low membranous EGFR expression as a reference

^b^Low nuclear EGFR expression as a reference

Note: Boldfaces as statistical significance

### Survival benefits of the combination of differentially located EGFR proteins

**Fig. 2:**
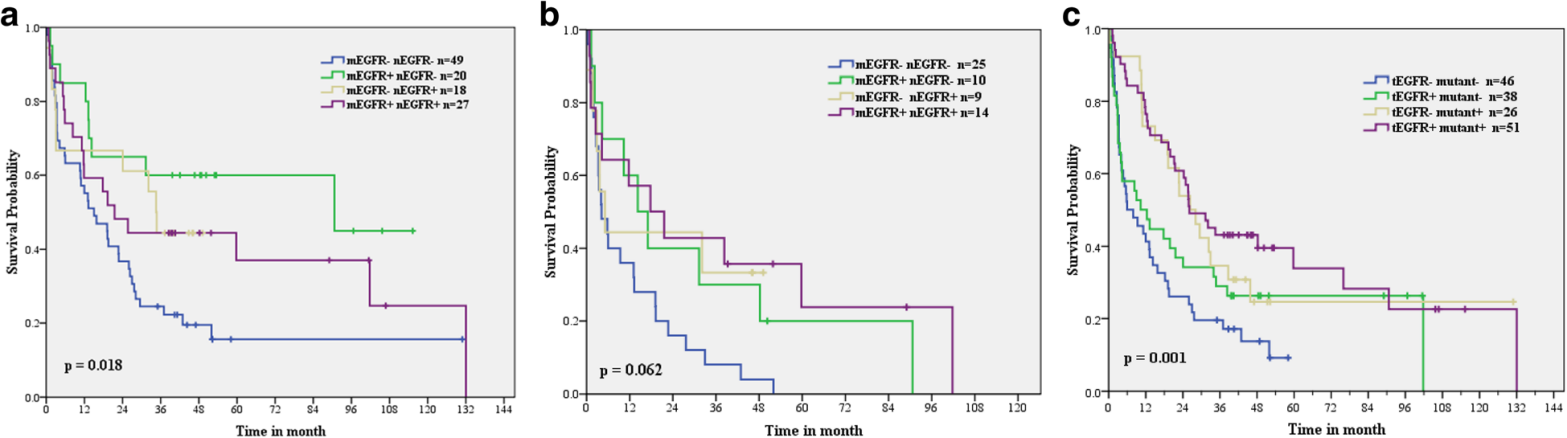
Survival analyses. **a** patients without brain metastasis and **b** patients with a history of smoking, stratified by high **m**EGFR and **n**EGFR expression; **c** total patients stratified by **t**EGFR expression and EGFR mutations

Since **n**EGFR proteins originate from the nuclear translocation of **m**EGFR proteins, we combined both immunostain types and divided them into four subgroups (**m**EGFR^−^ **n**EGFR^−^, **m**EGFR^+^ **n**EGFR^−^, **m**EGFR^−^ **n**EGFR^+^, and **m**EGFR^+^ **n**EGFR^+^), to investigate the synergistic effects on survival outcome. Although we did not find any survival differences among all patients (*p* = 0.112, data not shown), patients with low **m**EGFR and **n**EGFR expression had the lowest survival rate among patients without brain metastasis and with a history of smoking (*p* = 0.018 and 0.062, respectively, Fig. 2a and b).

Next, we considered high **m**EGFR and **n**EGFR expression together as total EGFR (**t**EGFR) expression, defined as either high **m**EGFR or **n**EGFR expression, to investigate the treatment response in patients receiving different anti-cancer therapies. **t**EGFR expression was significantly associated with a reduced mortality risk in patients with a history of smoking and without brain metastasis (HR[95% CI] = 0.24[0.07–0.81], *p* = 0.013; HR[95% CI] = 0.43 [0.18–1.06], *p* = 0.045, respectively) who were receiving platinum-based chemotherapy, as well as in patients without brain metastasis (HR [95% CI] = 0.45[0.20–0.98], *p* = 0.040) after EGFR TKI use. All data are shown in Table 4.

**Table 4:** Comparisons of treatment responses according to **t**EGFR expression

Parameters	No	**t**EGFR	Median^a^ (m)	HR (95%CI)	*P* value
Smokers					
Platinum	9	Positive	59.8	0.24 (0.07~ 0.81)	**0.013**
	8	Negative	20.9	1	
TKIs	9	Positive	32.1	0.40 (0.12~ 1.33)	0.122
	6	Negative	16.2	1	
Radiation	9	Positive	17.0	0.56 (0.21~ 1.53)	0.253
	11	Negative	13.1	1	
No brain metastasis					
Platinum	18	Positive	59.8	0.43 (0.18~ 1.06)	**0.045**
	14	Negative	23.4	1	
TKIs	20	Positive	33.3	0.45 (0.20~ 0.98)	**0.040**
	16	Negative	17.5	1	
Radiation	14	Positive	58.2	0.55 (0.20~ 1.48)	0.230
	11	Negative	12.3	1	

^a^Median = median survival time

Note: Boldfaces as statistical significance

### Synergistic effect of tEGFR protein and EGFR mutations on overall survival

Based on lung adenocarcinoma patients with EGFR mutations responsible to EGFR TKIs, we compared the combined effects of **t**EGFR protein and EGFR mutations on clinical benefits. Univariate analysis had shown the survival difference (*p* = 0.001, Fig. 2c) in four subgroups (**t**EGFR^**−**^ mutant^**−**^, **t**EGFR^+^ mutant^**−**^, **t**EGFR^**−**^ mutant^+^ and **t**EGFR^+^ mutant^+^). Then we examined the treatment response to platinum-based chemotherapy and found that comparing with patients without any biomarker (**t**EGFR^**−**^ mutant^−^), **t**EGFR protein was significantly associated with low mortality risk (HR[95% CI] = 0.33[0.12~ 0.92], *p* = 0.029; adjusted HR[95% CI] = 0.36[0.13~ 1.02], *p* = 0.055, with the use of TKI). All data are shown in Table 5.

**Table 5:** Hazard ratios for overall survival in the joint subgroups with platinum-based chemotherapy

**t**EGFR/mutant	Number	Median (m)^a^	Unadjusted HR (95% CI)	*p*	Adjusted HR^b^ (95%CI)	*p*
**t**EGFR^−^ mutant^−^	11	18.2	1.0		1.0	
**t**EGFR^+^ mutant^−^	10	34.7	0.33 (0.12~ 0.92)	**0.029**	0.36 (0.13~ 1.02)	0.055
**t**EGFR^−^ mutant^+^	10	29.5	0.65 (0.41~ 1.04)	0.070	0.64 (0.39~ 1.05)	0.076
**t**EGFR^+^ mutant^+^	21	25.9	0.74 (0.56~ 0.97)	**0.033**	0.83 (0.59~ 1.15)	0.260

Cox proportional model

^a^Median (m) = median survival time (month)

^b^Adjusted hazard ratio with TKI

Note: Boldfaces as statistical significance

## Discussion

This study investigated differentially located EGFR expression in lung adenocarcinoma. Our data indicate that high **m**EGFR expression is a more favorable prognostic factor in older patients, those with a history of smoking, and those without brain metastasis. Moreover, high **n**EGFR expression predicts early relapse in patients with distant metastasis. Notably, the combination of **m**EGFR and **n**EGFR expression is associated with survival benefits and with a more favorable response to anti-cancer therapies in patients with a history of smoking and without brain metastasis. Therefore, we suggest that differentially located EGFR expression synergistically predict survival outcomes and treatment responses in lung adenocarcinoma patients.

In this study, a high number of patients with low nodal stage exhibited high **m**EGFR expression, possibly indicating the initial stage of lung carcinogenesis. These results are different from those obtained in previous studies, which have reported a higher prevalence of EGFR overexpression in tumors of advanced stage and with lymph node invasion in colon and pancreatic cancer as well as in early stage (IA to IIIA) NSCLC [5, 20, 21]. Nevertheless, we did not observe any clinical associations for **n**EGFR proteins, although **n**EGFR has been associated with higher-stage breast cancer and higher disease stage in early-stage NSCLC [14, 19]. Such differences might have been a result of most enrolled patients having advanced-stage lung adenocarcinoma.

In accordance with recent studies on breast, ovarian and head-and-neck cancers, which have reported the prognostic value of **n**EGFR proteins for survival outcomes [12, 13, 19], the role of **n**EGFR expression in predicting recurrence risk in the metastasis subgroup was addressed in this study. However, clinicians may provide multi-agent therapies to patients with lung cancer relapse; therefore, the survival outcomes in patients with metastasis exhibited no differences. Altogether, we suggest that adjusting clinical management according to **n**EGFR expression at initial diagnosis might reduce early recurrence risk in patients with advanced lung adenocarcinoma.

In contrast to previous studies that EGFR overexpression has been associated with poor survival prognosis [5, 6, 22], this study has determined the survival benefits of differentially located EGFR proteins in those who had a history of smoking and no brain metastasis by observing more favorable treatment responses in patients with **t**EGFR expression. Although we could not exclude the effects of EGFR mutations on anti-EGFR therapies, we had found that patients with **t**EGFR protein was responsible to platinum-based chemotherapy regardless of EGFR mutations by observing the **t**EGFR^+^ mutant^**−**^ subgroup with significantly less mortality risk than the **t**EGFR^−^ mutant^**−**^ subgroup. Our findings are in accordance with a recent Chinese study that IHC positive **m**EGFR expression is associated with responses to EGFR TKIs in NSCLC patients with wildtype EGFR status [23]. Targeting EGFR protein has been reported to be an important treatment option for NSCLC [24]; therefore, EGFR proteins might be an indicator for treatment responses in patients with lung adenocarcinoma. However, the true mechanism warrants further investigation.

## Conclusions

This present study indicated that differentially located EGFR proteins might serve as a molecular marker of survival outcomes in patients with lung adenocarcinoma. Since EGFR proteins were responsible to platinum-based chemotherapy, treatment selection according to EGFR expression might be essential in the treatment of lung adenocarcinoma patients. Prospective studies are required to validate our theory.

## Additional file

Additional file 1 : **Table S1.** Distribution of differentially located EGFR expression in lung adenocarcinoma and **Table S2.** Morphologic characteristics for **m**EGFR expression, **n**EGFR expression and EGFR mutations in lung adenocarcinoma. (DOCX 16 kb)

## Abbreviations

CI: Confidence interval
EGFR: Epidermal growth factor receptor
HR: Hazard ratio
IHC: Immunohistochemistry
NSCLC: Non-small cell lung cancer
OR: Odds ratio
OS: Overall survival
PFS: Progression-free survival
TKI: Tyrosine kinase inhibitor
