# Supplementary material for: Prognostic value of localization of epidermal growth factor receptor in lung adenocarcinoma
Source: J Biomed Sci. 2018 Jun 28;25:53. doi: 10.1186/s12929-018-0451-3 (PMC6022489; doi:10.1186/s12929-018-0451-3)
Supplement: Supplementary file 1 — : Table S1. Distribution of differentially located EGFR expression in lung adenocarcinoma and Table S2. Morphologic characteristics for mEGFR expression, nEGFR expression and EGFR mutations in lung adenocarcinoma. (DOCX 16 kb) [file 12929_2018_451_MOESM1_ESM.docx]

**Table S1** Distribution of differentially located EGFR expression in lung adenocarcinoma

| Parameter |  | **High nuclear EGFR** | |  | *P* value |
| --- | --- | --- | --- | --- | --- |
|  |  | **Negative** | **Positive** | **Total** |  |
| **High membranous EGFR** | **Negative** | 65 (40.37)^a^ | 27 (16.77) | 92 (57.14) | **0.0033** |
|  | **Positive** | 33 (20.50) | 36 (22.36) | 69 (42.86) |  |
|  | **Total** | 98 (60.97) | 63 (39.13) | 161 (100.0) |  |
| Chi-squared test | | | |  |  |
| ^a^ Data are presented as n (%) of total patients. | | | |  |  |

**Table S2** Morphologic characteristics for **m**EGFR expression, **n**EGFR expression and EGFR mutations in lung adenocarcinoma

| **Subtypes** | **No** | **mEGFR** | *P* value | **nEGFR** | *P* value | **EGFR mutations** | *P* value |
| --- | --- | --- | --- | --- | --- | --- | --- |
| **Acinar** | **79** | **41 (51.9%)** | **0. 128** | **36 (45.6%)** | **0.0193** | **43 (57.3%)^a^** | **0.0164** |
| **Solid** | **51** | **16 (31.4%)** |  | **10 (19.6%)** |  | **19 (39.6%)^b^** |  |
| **Papillary or micropapillary** | **14** | **6 (42.9%)** |  | **8 (57.1%)** |  | **10 (76.9%)^c^** |  |
| **Lepidic** | **8** | **4 (50.0%)** |  | **5 (62.5%)** |  | **4 (66.7%)^d^** |  |
| **Mucinous** | **4** | **0 (0%)** |  | **2 (50%)** |  | **0 (0%)** |  |
| **AIS^e^ nonmucinous** | **5** | **2 (40%)** |  | **2 (40%)** |  | **1 (20%)** |  |
| **^a^ 2 missing; ^b^ 3 missing ; ^c^ 1 missing ; ^d^ 2 missing ; ^e^AIS: Adenocarcinoma in situ** | | | | | | | |

**Chi-square test**
